# Brain structural and functional changes in patients with major depressive disorder: a literature review

**DOI:** 10.7717/peerj.8170

**Published:** 2019-11-29

**Authors:** Lisong Dai, Hongmei Zhou, Xiangyang Xu, Zhentao Zuo

**Affiliations:** 1 Department of Radiology, Liyuan Hospital, Tongji Medical College, Huazhong University of Science and Technology, Wuhan, China; 2 State Key Laboratory of Brain and Cognitive Science, Beijing MRI Center for Brain Research, Institute of Biophysics, Chinese Academy of Sciences, Beijing, China; 3 Sino-Danish College, University of Chinese Academy of Sciences, Beijing, China; 4 Center for Excellence in Brain and Science and Intelligence Technology, Chinese Academy of Sciences, Beijing, China

**Keywords:** Depression, Magnetic resonance imaging, Central execution network, Salience network, Brain network, Neuroimaging, Default network, fMRI, Functional connectivity, Functional magnetic resonance imaging

## Abstract

Depression is a mental disorder characterized by low mood and anhedonia that involves abnormalities in multiple brain regions and networks. Epidemiological studies demonstrated that depression has become one of the most important diseases affecting human health and longevity. The pathogenesis of the disease has not been fully elucidated. The clinical effect of treatment is not satisfactory in many cases. Neuroimaging studies have provided rich and valuable evidence that psychological symptoms and behavioral deficits in patients with depression are closely related to structural and functional abnormalities in specific areas of the brain. There were morphological differences in several brain regions, including the frontal lobe, temporal lobe, and limbic system, in people with depression compared to healthy people. In addition, people with depression also had abnormal functional connectivity to the default mode network, the central executive network, and the salience network. These findings provide an opportunity to re-understand the biological mechanisms of depression. In the future, magnetic resonance imaging (MRI) may serve as an important auxiliary tool for psychiatrists in the process of early and accurate diagnosis of depression and finding the appropriate treatment target for each patient to optimize clinical response.

## Introduction

Major depressive disorder (MDD) is a mental illness characterized by significant persistent low mood and emotional changes. Its clinical manifestations include depression, sorrow, anhedonia, rumination. Patients with severe depression may have suicidal will or behavior. According to statistics, depression has become the most widely distributed mental disorder in the globe (Smith, 2014), and it is also one of the diseases with the highest disability-adjusted life year (Global Burden of Disease Study, 2015). Risk factors for depression include adverse life events, external environmental stress, cognitive impairment, depressed parents, social dysfunction, and being female (Hammen, 2018). Patients with malignant tumors, diabetes, chronic physical pain, or cardiovascular and cerebrovascular diseases also had higher rates of depression than healthy controls (Bortolato et al., 2017; Hare et al., 2014; Kales, Maixner & Mellow, 2005; Réus et al., 2019; Sheng et al., 2017). Thus, both structural and functional disorders, especially in the brain, may contribute to depression. Complex structural connectivity supports various physiological and social functions of the brain, which can process a variety of information efficiently and accurately (Park & Friston, 2013). At present, electroencephalogram (EEG) is often used by clinicians in the diagnosis of depression. However, due to the inability of EEG to provide spatial information and relatively low specificity, the diagnostic value provided by this method is limited.

On the other hand, neuroimaging can compensate for the defect of EEG, providing more spatial information and locating abnormal brain areas in patients with depression (Keren et al., 2018). For now, neuroimaging studies have confirmed that major depressive disorder is closely related to brain structural and functional abnormalities (De Kwaasteniet et al., 2013; Korgaonkar et al., 2014). Magnetic resonance imaging (MRI) is a noninvasive, reproducible, and acceptable technique that can provide more biological information than EEG with higher spatial resolution.

Gray matter is a significant component of the central nervous system, and the volume of gray matter in the brain is associated with many physiological senses and higher functions, including muscle control, vision and hearing, memory, emotion, language, decision-making and self-control (Rogers & De Brito, 2016; Zatorre, Fields & Johansen-Berg, 2012). Volume changes of gray matter can be detected by processing structural MRI information with Voxel-based morphometry (VBM).

The primary function of white matter is to transmit information efficiently and accurately between different gray matter areas of the central nervous system. Reduced white matter connectivity and volume can lead to impaired information delivery, which may cause deficits in attention, declarative memory, executive function, and intelligence (Fields, 2008; Reddick et al., 2006). Diffusion tensor imaging (DTI) can contribute to the assessment of the structural connectivity of nerve fiber bundles by displaying structural connections (Basser, Mattiello & LeBihan, 1994)

On the other hand, the resting and active status of brain regions can be observed by detecting fluctuations in blood oxygen levels. Blood oxygenation level-dependent (BOLD) functional MRI (fMRI) can contribute to the assessment of brain abnormalities by showing changes in brain activity of subjects in resting-state or task-state. Especially, resting-state functional magnetic resonance has become an essential basis for brain functional analysis (Biswal et al., 1995). The comprehensive application of structural and functional imaging provides a possible way to elucidate the etiology and pathogenesis of depression.

This article reviews recent advances in neuroimaging studies related to depression and summarizes the imaging changes of the disease from the structural and functional aspects.

## Survey Methodology

**Figure 1:**
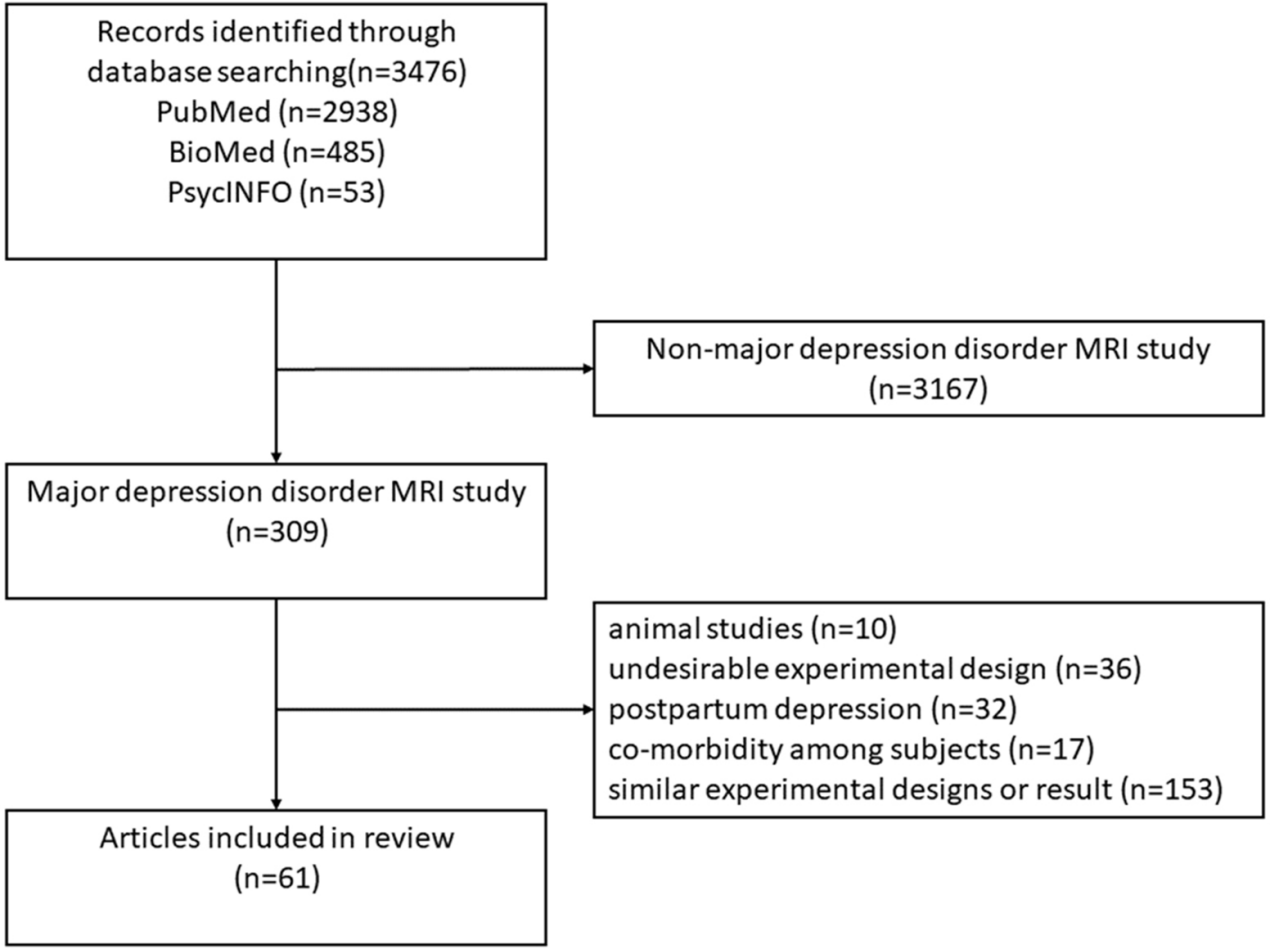
Flowchart of the decision tree.

Article searching was performed in PubMed , BioMed and PsycINFO between earliest record and September 1, 2019, using (“major depressive disorder” OR “unipolar depression” OR “depressive disorder, treatment-resistant”) AND (MRI OR “magnetic resonance imaging” OR VBM OR “Voxel-based morphometry” OR DTI OR “diffusion tensor imaging” OR fMRI OR “functional magnetic resonance imaging” OR BOLD or “blood oxygen level-dependent” OR “resting-state fMRI” OR “functional connectivity” OR rsfMRI OR “resting-state functional connectivity”) as search terms in title and abstract. Two authors jointly established inclusion and exclusion criteria and applied them to literature screening and quality assessment. These criteria were: (1) DSM-III, DSM-IV, DSM-V, ICD-10 or ICD-11 was used as criteria for diagnosing depression; (2) The experimental group and the control group matched at the age and gender level; (3) Clearly grouped medicated and unmedicated patients instead of mixing them into one group; (4) Subjects in the experimental group were not accompanied by bipolar disorder or other mental or organic diseases; (5) Studies with fewer than 10 people in the experimental group were excluded; (6) Studies on postpartum depression was excluded; (7) Animal experiments were excluded. The process of literature selection is shown in the flowchart (Fig. 1). Participant information and results from MRI studies are showed in the Supplemental Information.

## Brain Structural Abnormality in Depression

### Gray matter changes

VBM is widely used in the study of abnormal brain anatomy. This technique uses the statistical parameter map method to measure the volume and density of each voxel corresponding to gray matter and white matter. Changes were quantitatively calculated to assess changes in gray matter and white matter.

Meta-analyses indicate that hippocampal volume reduction is the most common brain anatomical change in patients with depression (Arnone et al., 2012; Cole et al., 2011), and, especially, the atrophy is most pronounced in the cornu ammonis, dentate gyrus, and subiculum (Roddy et al., 2019). As an essential part of the limbic system, the hippocampus plays a vital role in memory processing and emotional management. Moreover, studies (Buddeke et al., 2017; Den Heijer et al., 2011) found that depressive symptoms and hippocampal atrophy are mutually reinforcing and aggravating. Also, the volume of the cingulate cortex, another part of the limbic system associated with memory and mood formation, was smaller in depressed patients than in healthy controls (Rodriguez-Cano et al., 2014; Wise et al., 2017).

Frontal atrophy is also one of the critical changes in depression (Grieve et al., 2013). Studies have shown that the medial prefrontal cortex, frontal cortex, dorsolateral prefrontal cortex atrophy is particularly significant (Bludau et al., 2016; Van Eijndhoven et al., 2013; Zhao et al., 2014). The frontal cortex plays an important role in emotional cognition and working memory (Bludau et al., 2014).

In addition to the frontal lobe, the volume of the bilateral putamen and left thalamus in patients with depression is also smaller in contrast to healthy controls (Lu et al., 2016). These gray matter nuclei are related to memory, information transmission, and emotional management. Also, the degree of atrophy of the amygdala is positively correlated with the severity of depressive symptoms in patients (Zhang et al., 2016). And meta-analysis showed that patients with depression who had a larger gray matter before treatment were also better treated with medication (Fonseka, MacQueen & Kennedy, 2018).

### White matter changes

Abnormal white matter is also widespread in patients with depression (Liao et al., 2013). DTI shows the location and direction of the white matter bundle. Tract-based spatial statistics (TBSS) can quantitatively measure the fraction anisotropy (FA) of the nerve white matter fibers, and compare the white matter bundle skeletons of different subjects to locate the microstructure abnormalities of the brain white matter accurately (Smith et al., 2006).

Meta-analysis (Jiang et al., 2017a) indicated that the FA values in the corpus callosum, white matter in the right cerebellar hemisphere and bilateral superior longitudinal plasma of depressed patients were significantly lower than those in the healthy control group, and it was indicated that the abnormality of the corpus callosum was particularly prominent (Han et al., 2014). And Studies (Cole et al., 2012; De Diego-Adelino et al., 2014) showed that the extent of the FA decline in the corpus callosum, and bilateral upper longitudinal was positively correlated with the severity of depressive symptoms and duration of onset. And patients with suicide attempt history had lower FA in the dorsomedial prefrontal cortex than those without suicide attempt history and healthy controls (Olvet et al., 2014). Not only that, but the lower FA value in the ventral medial prefrontal area is more pronounced in patients with refractory depression (De Diego-Adelino et al., 2014). The decrease in the FA value of the superior frontal gyrus, superior longitudinal fasciculus, and corpus callosum can even predict the depression in the elderly (Reppermund et al., 2014). A study (Henderson et al., 2013) of adolescents with depression found that patients with more severe depressive symptoms had a greater FA reduction in sagittal stratum, anterior thalamic radiation, genu of the corpus callosum and anterior cingulate near the precuneus. At present, there are still many inconsistencies in the study on the abnormal white matter fiber bundles in depression. In the future, multi-site large sample studies can be carried out to verify the above research results.

### Cerebrovascular changes

On the other hand, compared with the healthy control group, elderly patients with depression have more severe cerebral vascular lesions such as white matter hyperintensities (WMH), subcortical lacunar, microinfarction, and microangiopathy (Wang et al., 2014). As early as 1997, Alexopoulos (Alexopoulos et al., 1997) proposed the “vascular depression hypothesis”, which believes that cerebrovascular disease and its subsequent white matter changes are an essential part of the pathogenesis of late-onset depression. A recent meta-analysis (Van Agtmaal et al., 2017) indicates that white matter hyperintensities are significantly associated with the incidence of depression. These white matter lesions are considered to be significant predictors of late-onset depression (Park et al., 2015), of which subcortical white matter lesions are strictly related to the severity of depressive symptoms and cognitive impairment (Taylor, Aizenstein & Alexopoulos, 2013). Besides, patients with depression with severe changes in leukoencephalopathy have worse symptoms and cognitive function after antidepressant treatment (Sheline et al., 2010).

## Brain Functional Abnormality in Depression

fMRI is widely used in the study of abnormal brain activity. When the neuronal activity is enhanced, the local blood flow in the cortex of the functional brain area is significantly increased, and the oxygen consumption is relatively insignificant, resulting in the proportion of deoxygenated hemoglobin/oxyhemoglobin is reduced. Due to deoxygenated hemoglobin is a paramagnetic substance, the functional region shows a different BOLD signal compared with the inactive brain region. Neuronal activity in a resting state or giving emotional stimuli and cognitive tasks can be indirectly reflected by a BOLD signal representing the local neuron activity of the brain.

### Regional brain activity changes

Depending on the purpose and the experimental design of the study, the fMRI study can be divided into two types: resting-state and task-based. The former mainly examines the spontaneous nerve activity of the subject in a calm and awake state, while the latter mainly explores the activity state of the brain when the individual is subjected to emotional stimulation or completing specific tasks.

A study (Hamilton et al., 2012) indicated, compared to the control group, MDD patients showed increased resting activity in the pulvinar, which is an essential nucleus in the thalamus and is thought to be functionally synchronized with nodes in the salient network, such as the amygdala, the insular lobes, and the anterior cingulate gyrus. Thus, it may enhance the response to negative emotional information in the salience network. Also, a meta-analysis (Kuhn & Gallinat, 2013) showed, at resting state, increased activity in the ventral medial prefrontal cortex, the left ventral striatum, and left thalamus and decreased activity in the left postcentral gyrus, left fusiform gyrus and left insula relative to controls in patients with depression.

On the other hand, due to the differences in experimental task design and sample selection methods, although many task-based fMRI experiments have studied the abnormalities of brain activity patterns in patients with depression during cognitive and emotional processing, consistent conclusions are still lacking (Muller et al., 2017). A meta-analysis (Miller et al., 2015) of adolescent depression found that hyperactivity of the anterior cingulate gyrus and thalamus may lead to depression patients being highly sensitive to emotional stimuli, while anhedonia may be caused by hypoactivation of the cuneus and posterior insula during reward processing. Interestingly, in depressed patients, the amygdala showed a “dual-separation” pattern of hyperactivity in response to negative stimuli (Tao et al., 2012) and decreased response to positive stimuli (Suslow et al., 2010). Likewise, in a small sample reward study (Takamura et al., 2017), MDD patients did not experience increased striatal activity in response to reward stimuli as healthy controls did.

Young et al.’s small sample studies (Young et al., 2017a; Young et al., 2017b) found that after real-time functional magnetic resonance imaging neurofeedback training, the amygdala activity of depressed patients could be relatively restored to normal, and patients’ depressive symptoms were reduced and their ability to recall positive memories was improved. A study (Holmes & Pizzagalli, 2008) indicates that the decline in cognitive ability in patients with depression is caused by the distribution of excessive neural pathway resources in negative consciousness and rumination. A small sample study by Liao et al. (2012) showed that patients with depression had a bias in their perception of pleasure and neutral stimuli, which was associated with abnormal activity in the bilateral amygdala and the right dorsolateral prefrontal cortex.

### Brain network functional connectivity changes

**Figure 2:**
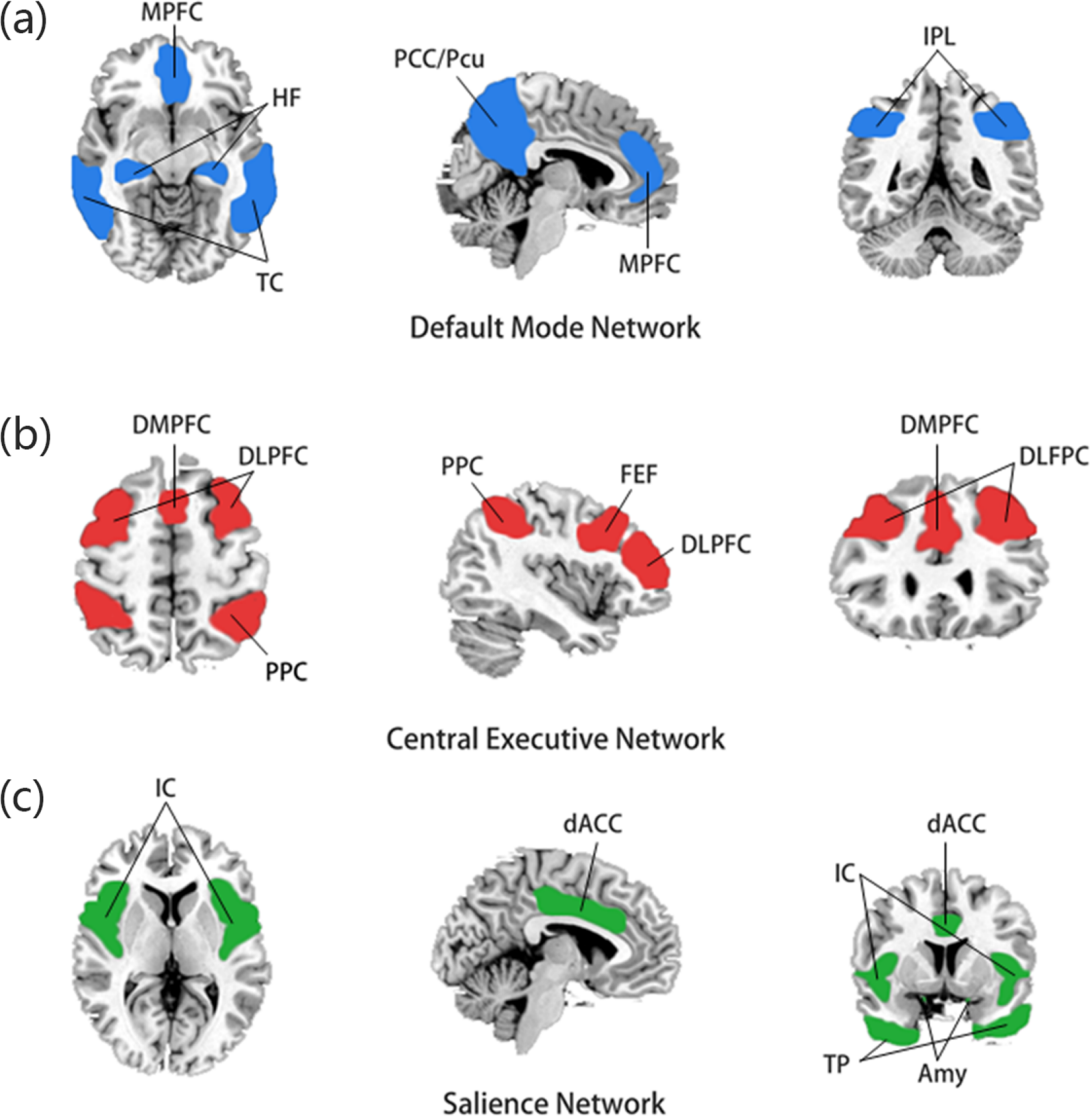
Components of the triple network model. (A) The default mode network is mainly composed of the medial prefrontal cortex (MPFC) and posterior cingulate cortex/precuneus (PCC/PCu), and the temporal cortex (TC), hippocampus formation (HF) and inferior parietal lobule (IPL) are also closely related to this network. (B) The central executive network (CEN) is mainly composed of the dorsolateral prefrontal cortex (DLPFC) and posterior parietal cortex (PPC), dorsolateral prefrontal cortex (DMPFC) and frontal eye field (FEF). (C) The salience network is composed of the insular cortex (IC), dorsal anterior cingulate cortex (dACC), temporal pole (TP) and amygdala (Amy).

Functional connectivity is defined as the temporal correlation of multiple brain regions. Each brain region, defined as a node, is connected, eventually forming a brain network with highly complex and concentration. These brain networks play an important role in cognitive and emotional processing (Park & Friston, 2013). In 2011, Menon (Menon, 2011) proposed the “triple network model” theory, which concluded that abnormal functional connectivity of the default mode network (DMN), the central execution network (CEN), and the salience network (SN) (Fig. 2) is closely related to various mental illnesses, including depression. Not only that, by examining abnormalities in brain network functional connectivity in 711 depressed patients, Drysdale et al. (2017) defined four neurophysiological subtypes of depression and to some extent, successfully predicted their rTMS treatment effect.

The most common methods for studying brain function connectivity include seed-based correlation analysis (SCA) and independent component analysis (ICA). SCA predetermines “seed” (a region of interest) based on previous assumptions, and calculates the correlation with the other voxels or specific other regions of the brain by their BOLD signal fluctuations. In contrast, ICA uses all available data in the fMRI image and decomposes it several independent components. An increase in functional connectivity represents increased synchronization between the two regions.

#### Default mode network

The DMN consists mainly of the medial prefrontal cortex, posterior cingulate cortex/precuneus, and inferior parietal lobule (Raichle, 2015). Also, the hippocampal formation and the temporal cortex are thought to be closely related to the DMN (Raichle, 2015). The default network is usually active when a person is at rest, immersed in self-reflection, memory, and imagining the future. In some studies, the default mode network can be subdivided into an anterior sub-network and a posterior sub-network.

The default mode network is currently the most commonly studied brain network for depression. So far, one of the most consistent conclusions is that the connectivity of multiple nodes in the default mode network of depression patients is abnormally increased (Posner et al., 2013), and is considered to be closely related to the patient’s rumination symptom (Hamilton et al., 2015). Moreover, after treatment with antidepressants, the functional connectivity abnormality in the posterior network was restored, but the anterior connectivity abnormalities persisted (Li et al., 2013), and it is believed that the latter may be an important cause of higher recurrence rate of depression. And this “dissociative pattern” is also found in a study by Guo et al. (2014), in which it was suggested increased network homogeneity in the anterior DMN but decreased in the posterior one. In addition, many studies (Connolly et al., 2013; Greicius et al., 2007) found that though anterior cingulate cortex is not the central node of default mode network in healthy controls, at resting state, functional connectivity between anterior cingulate cortex and other nodes within default mode network is significantly enhanced in depressed patients. And this “over-recruitment” feature indicates that the anterior cingulate cortex of the depressed patients may be abnormally involved in the default mode network, which is considered to be unique to major depressive disorder (Menon, 2011). Moreover, children at familial risk for depression also exhibited greater functional connectivity between the default mode network and subgenual anterior cingulate cortex (Chai et al., 2016), suggesting that the abnormal default mode network connectivity may have occurred early in the onset of illness.

#### Central executive network

The CEN, also known as the executive control network, consists mainly of the dorsolateral prefrontal cortex, the dorsal anterior cingulate cortex, the posterior parietal cortex, and the frontal eye field and plays a role in working memory, problem-solving, goal-oriented behavior and decision-making.

Compared with the control group, the functional connectivity between the central executive network and the default mode network of MDD patients is decreased, while the functional connectivity with the salience network is increased, which might be related to the rumination (Jiang et al., 2017b). In addition, the internal connectivity within the central executive network of MDD patients is also decreased than that of the control group, and, in particular, the dorsolateral prefrontal cortex showed the most significant decline in functional connectivity with other nodes in the network (Liston et al., 2014), which is considered to be closely related to the patients’ depression symptoms and maladaptive mood regulation (Alexopoulos et al., 2012).

#### Salience network

The SN consists of the insular cortex, dorsal anterior cingulate cortex, temporal pole and amygdala and is responsible for detecting and filtering stimuli, as well as in recruiting relevant functional networks (Menon & Uddin, 2010). Completing a variety of complex functions, including communication, social behavior, and self-awareness.

**Figure 3:**
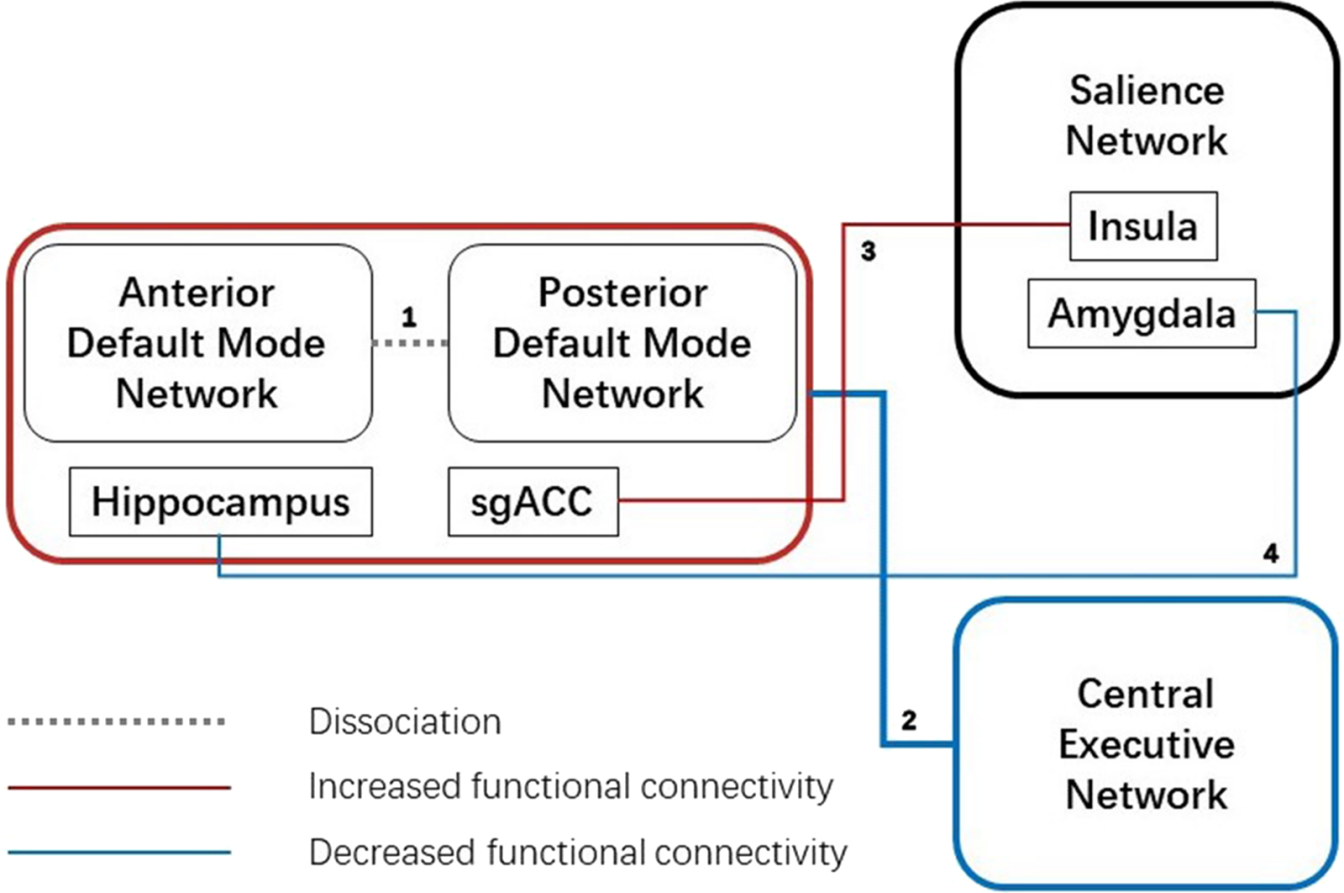
Aberrant functional connectivity between three networks. 1. Dissociation between anterior and posterior default mode network; 2. Decreased functional connectivity between the default mode network and central executive network; 3. Increased functional connectivity between sgACC (subgenual anterior cingulate cortex), which is “over-recruited” in default mode network, and insula, a key node in the salience network; 4. Decreased functional connectivity between the hippocampus, which is functionally closely related to the default mode network, and amygdala, another vital node in the salience network.

Abnormal salience network connectivity is considered to be one of the crucial links in the pathogenesis of depression, especially in the insula and amygdala. Salience network, especially the right anterior insula, is thought to be critical in the transition from the central execution network’s dominant “execution state”. to the default state-preferred “default state” (Goulden et al., 2014). A study (Connolly et al., 2013) found elevated connectivity between the subgenual anterior cingulate cortex and insula, which may result in enhanced functional connectivity between the default mode network and the salience network, thus hindering the above transition (Fig. 3).

Another important anomaly node in the salience network is the amygdala. In adults and adolescents with depression and children at high risk of depression, the functional connectivity between the amygdala and the hippocampus is found to be decreased (Cullen et al., 2014; Luking et al., 2011; Zeng et al., 2012), while hyperactivity in the amygdala, as found in brain activity studies (Liao et al., 2012; Tao et al., 2012), is considered to be a compensation mechanism for this weak functional connectivity. Also, increased functional connectivity between the amygdala and subgenual anterior cingulate cortex is thought to be associated with long-term negative emotions in patients (Davey et al., 2015). Moreover, functional connectivity between the amygdala and the brainstem and precuneus are reduced in depressed patients compared with controls (Zhang & Li, 2012).

## Conclusion

In summary, neuroimaging studies have shown that depression involves multiple brain regions with structural and functional abnormalities, most of which are related to the limbic system, the default mode network, the central execution network, and the salience network. Together, they caused a variety of clinical symptoms of depression. Among them, atrophy and abnormal activity of parahippocampal gyrus and hippocampus led to patients’ positive memory recall disorder, which may further lead to anhedonia. Negative emotions and exaggerated responses to negative stimuli and degrading life events were mainly related to amygdala activity abnormality. While the decrease in the performance of cognitive processing and working memory is mainly related to the decrease in CEN functional connection, the abnormality of SN is mainly due to the abnormal adjustment of functional balance between DMN and CEN. However, due to the low consistency and reproducibility of the study results and the lack of clinical specificity at the individual level, the above examination methods have not been widely used in clinical diagnosis. Future research needs to enhance the homogeneity of the sample and obtain more data from patients of different age groups, different symptoms and related diseases to obtain highly specific results. It is also worthwhile to look for the commonality of the brain structure and/or brain function of patients in various subgroups and to find the best treatment.

##  Supplemental Information

10.7717/peerj.8170/supp-1 Table S1 Demographics and results of the studies included in the article * prospective study

## Additional Information and Declarations

### Data Availability

The following information was supplied regarding data availability: Subject information and study results of depression-related MRI studies included in this paper are available as a Supplemental File.
